# Friendship Selection and Influence Processes for Physical Aggression and Prosociality: Differences between Single-Sex and Mixed-Sex Contexts

**DOI:** 10.1007/s11199-017-0818-z

**Published:** 2017-09-13

**Authors:** Jan Kornelis Dijkstra, Christian Berger

**Affiliations:** 1 Department of Sociology and Interuniversity Center for Social Science Theory and Methodology (ICS), University of Groningen, Grote Kruisstraat 2/1, 9712 TS Groningen, The Netherlands; 2 Department of Psychology, Pontificia Universidad Católica de Chile, Santiago, Chile

**Keywords:** Physical aggression, Prosociality, Stochastic-actor based modeling (RSIENA), Same-sex/mixed-sex contexts, Social networks, Selection, Influence, Perceived popularity

## Abstract

The present study examined to what extent selection and influence processes for physical aggression and prosociality in friendship networks differed between sex-specific contexts (i.e., all-male, all-female, and mixed-sex classrooms), while controlling for perceived popularity. Whereas selection processes reflect how behaviors shape friendships, influence processes reveal the reversed pattern by indicating how friends affect individual behaviors. Data were derived from a longitudinal sample of early adolescents from Chile. Four all-male classrooms (*n* = 150 male adolescents), four all-female classrooms (*n* = 190 female adolescents), and eight mixed-sex classrooms (*n* = 272 students) were followed one year from grades 5 to 6 (*M*_age_ = 13). Analyses were conducted by means of stochastic-actor-based modeling as implemented in RSIENA. Although it was expected that selection and influence effects for physical aggression and prosociality would vary by context, these effects showed remarkably similar trends across all-male, all-female, and mixed-sex classrooms, with physical aggression reducing and with prosociality increasing the number of nominations received as best friend in all-male and particularly all-female classrooms. Further, perceived popularity increased the number of friendship nominations received in all contexts. Influence processes were only found for perceived popularity, but not for physical aggression and prosociality in any of the three contexts. Together, these findings highlight the importance of both behaviors for friendship selection independent of sex-specific contexts, attenuating the implications of these gendered behaviors for peer relations.

**Electronic supplementary material:**

The online version of this article (10.1007/s11199-017-0818-z) contains supplementary material, which is available to authorized users.

Early adolescence is a critical time in the development of friendships (Steinberg 2007). Friends become more important for the provision of emotional support (Bukowski and Sippola 2005), identity development (Bagwell and Smith 2011), and bridging individuals’ experiences with participation in a wider peer culture (Espelage et al. 2007). Friendships are also important for adolescents’ engagement in and development of behaviors (Brechwald and Prinstein 2011). The interplay between the development of behaviors and friendships captures two fundamental processes in adolescents’ peer contexts: friends’ influence and friendship selection. Influence suggests that friendships shape adolescents’ individual behaviors. Selection implies that adolescents change their friendships in accordance with their behaviors (or characteristics), also referred to as selection-similarity (Veenstra et al. 2013). In addition, selection processes encompass the effect of behaviors (or characteristics) on being nominated as a friend (capturing attractiveness, known as alter effects), and on nominating friends (capturing activity, known as ego-effects). Together, these processes tend to result in the same phenomenon: befriended adolescents are likely to be similar to each other in behaviors and characteristics— among those aggression (Dijkstra et al. 2011; Sijtsema et al. 2010a, b) and prosocial behavior (Logis et al. 2013).

Until now, most studies have focused on the relative contributions of selection and influence processes to similarity among affiliated peers. However, these processes are likely to depend on characteristics of the individuals, peers, relations, and contexts (Brechwald and Prinstein 2011). Research on these moderating effects for selection and influence might not only help to unravel inconclusive and, sometimes, contradictory findings, but also advance our knowledge of under what conditions adolescents are more or less likely to be influenced by or feel attracted to peers and selecting them as friends. For instance, at the individual level Rulison et al. (2013) showed that rejected adolescents were less likely to select aggressive peers, whereas Molano et al. (2013) found that a hostile attributional bias strengthened the influence of friends on adolescents’ individual aggression. At the contextual level, some studies showed that class-level status norms affected the strength of influence processes in classrooms regarding attitudes towards risk behaviors (Rambaran et al. 2013) and aggression (Laninga-Wijnen et al. 2016).

However, these studies are still scarce, and to date no known study has addressed the role of the gendered peer culture on peer selection and influence, over and above simply controlling for gender differences. This is surprising because one of the most important features steering early adolescents’ friendships is gender (Maccoby 2002; Veenstra et al. 2013). Early adolescents have a strong preference for same-gender peers as friends (Bagwell and Smith 2011; Dijkstra et al. 2011; Johnson 2004; Maccoby 2002), resulting in largely gender-segregated peer networks (Lam et al. 2014; Maccoby 1998; Mehta and Strough 2009). Selection effects for gender, however, might interact and be confounded with other attributes. The findings of earlier studies have suggested that physical aggression and prosocial behavior are gender-specific behaviors, with physical aggression being more prevalent among boys (Card et al. 2008; Crick and Grotpeter 1995; Martin and Halverson 1981) and prosocial behavior being consistently associated with girls (Rose and Rudolph 2006).

Questions are raised about what happens with the roles of these behaviors in processes of friendship selection and influence when adolescents are placed in a context with only same-gender peers. Do some behaviors become more prominent and others less important? How does the development of behaviors depend on the gendered nature of the setting in which they unfold? To explore these questions, we examined to what extent selection and influence processes for physical aggression and prosociality in friendship networks differ between classrooms with only male, only female, and both male and female students.

## Physical Aggression and Prosociality

During early adolescence young people gradually shift their attention from parents toward peers to establish close relationships and build a sense of belonging (Corsaro and Eder 1990; Giordano 2003; Ojanen et al. 2005). Peer relations become significant determinants of adolescents’ social and mental development (Rubin et al. 2009). In this sense, early adolescents’ selection of peers with whom they affiliate and risks of being socially excluded or the pressure for accepting peers as friends, even if not preferred, becomes more important (Sijtsema et al. 2010b). At the same time, processes of conformity to peer norms and imitation of behaviors to enhance the chances of acceptance become more salient (Wright et al. 1986). Hence, peer influence processes become more likely because adolescents may perceive the need to meet behavioral standards for peer approval and acceptance. This might be particularly salient for social behaviors such as physical aggression and prosociality; both have shown to be subject to selection and influence processes, although research findings are inconclusive (for an overview, see Veenstra et al. 2013).

Some studies have shown that early adolescents tend to become similar in their physical aggression to the peers with whom they associate (Berger and Rodkin 2012; Dishion and Tipsord 2011; Logis et al. 2013; Molano et al. 2013), although one study showed that this influence effect was stronger for instrumental and relational aggression than for physical aggression (Sijtsema et al. 2010a, b). Recently, Farrell et al. (2017) found a significant influence of peer pressure for fighting and friends’ problem behavior on adolescents’ aggression. There is also evidence that adolescents tend to select their friends based on similarity in aggression (Rulison et al. 2013), although other studies did not find these effects (Logis et al. 2013; Molano et al. 2013). Studies on prosocial behavior also have revealed inconclusive findings, with some studies showing evidence for influence (Barry and Wentzel 2006; Berger and Rodkin 2012; Farrell et al. 2017; Logis et al. 2013) and selection processes (Logis et al. 2013), whereas others found no effects (Molano et al. 2013). Although physical aggression and prosociality have been subject to selection and influence processes, the roles of both behaviors may be confounded by other attributes that may also drive friends’ selection and influence, such as gender.

## Friendships within Gender-Specific Contexts

Gender differences have been shown to influence social adaptation, particularly by the degree of rigidity in gender schemas or gender-typing. Rose and Rudolph (2006) conclude that gender differences can be observed both in the structure and in the content of children’s and adolescents’ relationships. They propose that emotional and behavioral adjustment is associated with gender differences and particularly with exposure to same- or cross-gender relationships. From a different perspective, Lurye et al. (2008) argue that the rigidity in gender-typing can be accompanied by poorer social adjustment because children and adolescents would miss opportunities for developing certain skills that are considered to be attributes of the other gender. In this line, Faris and Felmlee (2011) concluded that patterns of individual aggression were more likely to depend on gender segregation and cross-gender relationships than on main gender differences. In particular, aggression declines in contexts were cross-gender friendships are common. However, most of these studies, even though referring to gender segregation, were conducted in mixed-gender schools when assessing gender differences.

The question is: What happens with behaviors in selection and influence processes if examined in single-sex (i.e., all-male and all-female) and mixed-sex contexts? Friendship selection and influence are partially driven by behaviors and characteristics that are normative and salient within the peer context. However, some behaviors are to a certain degree gender-specific, with physical aggression being linked to boys and prosociality being associated with girls (Card et al. 2008; Crick and Grotpeter 1995; Martin and Halverson 1981; Rose and Rudolph 2006). Consequently, the salience of these behaviors might differ in contexts with only male or with only female students.

Studies on peer relations have shown that these two behaviors have different implications for male and female adolescents. Oberle et al. (2010), for instance, found that prosocial behavior was associated with peer acceptance among girls but not among boys. Furthermore, they showed that antisocial behavior was negatively associated with peer acceptance for girls, but unrelated to peer acceptance among boys. A study by Velásquez et al. (2010) showed that being victimized was associated with relational aggression for boys but with physical aggression for girls. Moreover, this association was enhanced for girls in all-girls educational settings. The authors argued that aggression is associated with victimization only when it is non-normative in a specific context, emphasizing the importance of the gendered context for the consequences of individual behavior.

Also highlighting the impact of contextual variation in gender composition, Drury et al. (2013) studied how girls’ gender identity and pressure to conform to gender norms differed between all-girls and mixed-sex schools, showing that deviation from gender norms was only related to victimization in single-sex schools. Johnson and Gastic (2014) also found that gender-nonconforming students, and those who varied from the dominant gender norms in their schools, were more likely to be victimized. However, this risk decreased in single-sex schools for girls, where female gender norms do not necessarily resemble traditional gender stereotypes (Johnson and Gastic 2014). Finally, Hanish et al. (2005) found in a study among preschoolers and kindergarteners that girls (but not boys) who spent time with peers with more externalizing behaviors became more aggressive over time. They speculated that, because externalizing behaviors are not normative for girls, these relationships are more salient and may thus be more influential.

Together, the findings of these studies suggest that non-normative behavior has implications for early adolescents’ peer relations. If aggression is non-normative for female early adolescents, and prosociality for male early adolescents, we speculate that the roles of these behaviors in selection and influence processes will become more pronounced and strengthened in a sex-specific context. Gender-specific behaviors may decrease their relevance in sex-specific contexts where their function of differentiation from the other sex fades.

## Friendship and Perceived Popularity

The roles of physical aggression and prosocial behavior in friendship selection and influence need to be considered along with adolescents’ social status (i.e., perceived popularity). Adolescents perceived to be popular may set the bar regarding which behaviors and attributes are salient and valued within their peer group (Berger and Caravita 2016; Dijkstra and Gest 2015). Whereas perceived popularity, as an important social goal during adolescence, can shape peer affiliations, aggression and prosociality are useful tools for this means (Cillessen and Rose 2005; Dijkstra et al. 2009; Faris and Felmlee 2011). For instance, Dijkstra et al. (2011) found that the effect of physical aggression on friendship selection disappeared when gender and perceived popularity were taken into consideration, suggesting that aggression similarity between friends is a by-product of same-gender selection and similarity in perceived popularity. Logis et al. (2013) also found that adolescents were more likely to select friends based on similarity in perceived popularity rather than on aggression or prosocial behavior. Thus, perceived popularity appears to be an important confound in the prediction of friendships during adolescence (see also Dijkstra et al. 2013). Hence, we controlled for perceived popularity when examining the roles of aggression and prosociality in selection and influence processes.

## The Present Study

The present study extends earlier studies that have addressed the role of aggression and prosociality in friendship selection and friends’ influence by assessing these processes in distinct gendered contexts: all-male, all-female, and both male and female schools, while controlling for perceived popularity. Our main hypothesis is that physical aggression is more important for selection and influence processes in all-female classrooms, whereas prosociality is more important for selection and influence processes in all-male classrooms. Specifically, we expect that the effects of physical aggression on nominating peers as friend (ego-effect) (Hypothesis 1a), being nominated as a friend (alter-effect) (Hypothesis 1b), forming friendships with similar peers (selection-similarity) (Hypothesis 1c) as well as its role in friendship influence (Hypothesis 1d) are stronger in all-female classrooms than in all-male classrooms. For prosociality, we expect that the effects of prosocial behavior on nominating peers as friend (ego-effect) (Hypothesis 2a), being nominated as friend (alter-effect) (Hypothesis 2b), forming friendships with similar peers (selection-similarity) (Hypothesis 2c) as well as its role in friendship influence (Hypothesis 2d) are stronger in all-male classrooms than in all-female classrooms.

To test our hypothesis that non-conforming behavior (physical aggression among female adolescents and prosocial behavior among male adolescents) is more salient in selection and influence processes in gender-segregated contexts, we performed longitudinal social network analysis. This approach allows researchers to untangle selection and influence processes in a sophisticated manner by modeling changes in behaviors and relations simultaneously (Snijders et al. 2010).

Given that most of the literature arises from studies carried out in developed countries (Europe and North America), by featuring a sample from Santiago, Chile, the present study also offers evidence from an understudied population, testing the universality of peer processes. Although several studies from Latin American countries highlighted the peculiarities of gender roles (Drury et al. 2013; Velásquez et al. 2010), previous studies with Chilean adolescents have shown that peer processes in this particular population resemble what has been reported in the literature (Berger et al. 2015; Berger and Palacios 2014; Berger and Rodkin 2012; Dijkstra et al. 2011).

## Method

### Participants

Participants were part of a study on peer relations in metropolitan Santiago, Chile. In total, 612 fifth and sixth graders (301 fifth and 311 sixth graders; age range 10–12) from four elementary schools were included in the study; four classrooms were all-males (*n* = 150), four were all-females (*n* = 190), and eight were mixed-sex classrooms (*n* = 272). The single-sex classrooms were all located within single-sex schools. These schools were private but received a public subsidy; this is representative of the majority of the Chilean school population. All schools were average in terms of family income, and they were located in low-to-middle socioeconomic status neighborhoods. Active consent was received from all students and their parents. For 36 students, no information was available at Wave 1. They were coded as missing. Attrition analyses showed that participants who were only present at one wave did not differ in physical aggression, prosociality, or perceived popularity from their peers who participated in both waves.

### Procedure and Measures

Participants were surveyed from June to August of 2010 (middle of the academic year) and were re-assessed during the same months in 2011. Surveys were completed during regular class hours through group administration, taking 45 min per classroom. Measures, consent protocols, and procedures to protect the confidentiality and rights of all participants were approved by the Institutional Review Board of the local university and by the principals of the schools involved in our research. All data were gathered through within-classroom peer nominations. Participants could nominate up to six classmates for each question. In mixed-sex contexts respondents could nominate male and female classmates. All self-nominations were excluded. Note that the following categorizations for physical aggression, prosociality, and perceived popularity were aimed at adequately capturing the distribution of our variables in our sample with sufficient cases for each category.

#### Friendships

Participants were asked to nominate up to six classmates whom they considered as their best friends. Friendship nominations were used to assess classroom friendship networks using adjacency matrices, containing information on whether a best friend relation was absent (zero) or present (one). Subsequently, three overall adjacency matrices were constructed for all-male classrooms, all-female classrooms, and mixed-sex classrooms, including the classroom networks. Structural zeros between classroom networks were used to indicate that participants were not able to nominate peers from other classrooms.

#### Physical Aggression

Participants could nominate up to six classmates who best fit the descriptor “who fights a lot.” To control for the potential number of classmates that could nominate, the number of nominations received was divided by the number of classmates. Because RSIENA cannot deal with continuous measures as outcomes, these proportion scores were then transformed into four categories: 1 = 0; 2 = .01–.10; 3 = .11–.20; and 4 = ≥ .21.

#### Prosociality

Two peer nomination items were used to assess prosociality. Again, participants could nominate up to six classmates for “who cooperates” and “who is kind to others.” Proportion scores were calculated for each question and then summed, and divided by two. Subsequently, the following four categories were constructed: 1 = 0; 2 = 2 = .01–.10; 3 = .11–.20; and 4 = ≥ .21.

#### Perceived Popularity

Participants could nominate up to six classmates whom they considered to be “popular” and “unpopular” in their classroom. Again, proportion scores for each item were calculated as the number of nominations received over the number of classmates. Unpopular scores were then subtracted from popular scores. These scores were then *z*-standardized across the sample, a common procedure in calculating perceived popularity (Cillessen and Marks 2011). These *z*-scores were then categorized into six categories with .05 as cut-off points, yielding six categories: 1 ≤ −.11; 2 = −.10– -.06; 3 = −.05– -.01; 4 = .00–.05; 5 = .06–.10; and 6 = ≥ .11.

### Analytic Strategy

We examined selection and influence processes using stochastic actor-based modeling as implemented in RSIENA (Simulation Investigation for Empirical Network Analysis). RSIENA estimates an actor-based model for the co-evolution of networks and behaviors over time, allowing simultaneously testing the interplay between changes in the social networks and changes in individuals’ attributes over time (Snijders et al. 2007). The changes in network relations (making new friendships or breaking existing ones) reflect network dynamics, including selection effects. Conversely, changes in individual attributes reflect behavioral dynamics, including influence effects. The estimates of the model are obtained through an iterative simulation procedure within a Markov Chain Monte Carlo approach (Snijders et al. 2007). The model imputes likely developmental trajectories between time points with the information from Wave 1 taken as starting point. These estimates are based on transition probabilities between probable states in the state space of possible configurations of the combination of network and behaviors. Estimates indicate the probability of specific change patterns for both individual attributes and network relations given the observed data. The estimation of changes in the network and individual attributes are modeled simultaneously. In this way, the program enables testing of selection and influence effects while each is controlled for the other (Veenstra and Steglich 2012). The SIENA program allows for the inclusion of cases with missing data by minimizing their influence on the estimation of results (for a detailed description, see Huisman and Steglich 2008).

RSIENA analyses yield two types of parameters. First, parameters with regard to the network represent both structural network effects and changes in the network, reflecting selection effects. In the current study and as recommended (Snijders et al. 2010), we included three structural network effects: (a) *density*, the number of outgoing ties, and, therefore, the density of the network; (b) *reciprocity*, the extent to which friendship choices are reciprocated; and (c) *transitivity*, the tendency of individuals to be friends with the friends of their friends (transitive triplets).

Next to these network characteristics, selection effects for physical aggression, prosociality, and perceived popularity were estimated. *Ego effects* indicate the extent to which physical aggression, prosociality, and perceived popularity affect the number of best friend nominations given to peers. Conversely, *alter effects* indicate the extent to which physical aggression, prosociality, and perceived popularity affect being nominated as a friend by peers. The parameter *selection-similarity* (the ego x alter effect in SIENA) indicates whether adolescents with higher values on physical aggression, prosociality, and perceived popularity were likely to choose peers as friends who also had higher values on these variables. In mixed-sex classrooms, we additionally included the ego and alter effects for gender as well as the same-sex effect, indicating to what extent friendship choices are directed to same-sex peers.

The second type of estimates indicates the extent to which physical aggression, prosociality, and perceived popularity change over time, referred to as behavior dynamics. First, the *linear shape* effect indicates the overall response to high or low values on the dependent variable (here: physical aggression, prosociality, and perceived popularity). A negative parameter indicates that the majority of respondents scored below the mean. Second, the *quadratic shape* effect expresses a feedback effect of the variable on itself. A positive parameter indicates that responses tend to occur on the extreme ends of the scale, reflecting a self-reinforcing effect. A negative value suggests that responses are unimodally scattered around the group average, indicating a self-correcting effect (also see Snijders et al. 2010). Together, the linear and quadratic shape effects (referred to as behavioral tendencies) can be interpreted as a curvilinear function, independent of other effects or explanatory mechanisms. Third, the influence effect estimates whether adolescents whose friends had higher values on the dependent variable (physical aggression, prosociality, and perceived popularity) also developed higher values themselves over time (the average alter effect in SIENA). The analyses were conducted separately for the all-male classrooms, all-female classrooms, and mixed-sex classrooms. For each context, the networks were combined into one overall network with structural zeros between classrooms to indicate that participants were not able to nominate peers from other classrooms (Veenstra and Steglich 2012).

To examine the differences among these three contexts, we first examined to what extent differences emerged in the significance of effects between contexts, indicating the salience of predictors of changes in peer relations and behaviors. Second, we formally calculated to what extent differences between the estimates were statistically significant using a *F*-test with the following formulae: $\widehat{\upbeta}a-\widehat{\upbeta}a/\sqrt{SEa^2+{SEb}^2}$, which under the null-hypothesis of no effect has an approximating standard normal distribution.

## Results

In the following section we first present the descriptive statistics for physical aggression, prosociality, and perceived popularity, as well as the friendship networks. Subsequently, we discuss the correlations among physical aggression, prosociality, and perceived popularity. We present this information separately for all-male, all-female, and mixed-sex contexts. Finally, we present the results of the RSIENA analyses to test our hypotheses.

### Descriptive Statistics

In Table 1 descriptive statistics are given for (a) physical aggression, (b) prosociality, and (c) perceived popularity. No large differences were found between all-male, all-female, and mixed-sex classrooms; although, as expected based on gender-specific normative characteristics, at time 1 physical aggression was somewhat lower in all-female classrooms as compared to mix-sex and all-male classrooms, *F*(2573) = 3.28, *p* = .038, ηp^2^ = .007. No significant differences were found for prosociality or perceived popularity. Repeated measures ANOVA with time (within subjects) and classroom sex composition (between subjects) showed no significant main effect for aggression, *F*(1573) = .69, *p* = .406, classroom sex composition, *F*(2573) = 2.09, *p* = .290) or for the interaction, *F*(2573) = 2.53, *p* = .080. Regarding prosociality, same analyses showed no effect for prosociality, *F*(1573) = 1.45, *p* = .229, but a significant main effect for classroom sex composition, *F*(2573) = 13.09, *p* = .001, ηp^2^ = .044, and for the interaction, *F*(2573) = 9.97, *p* = .001, ηp^2^ = .034, were found. Post-hoc Bonferroni contrasts showed that mixed-sex classrooms had higher levels of prosocialilty as compared to both all-male (*p* = .001) and all-female (*p* = .017) classrooms. Same repeated measures ANOVA analysis for perceived popularity showed no main effect for popularity, *F*(1573) = .77, *p* = .381, classroom sex composition, *F*(2573) = .78, *p* = .461, or for the interaction, *F*(2573) = 2.50, *p* = .083.

**Table 1:** Descriptive statistics for physical aggression, prosociality, and perceived popularity

	Classroom sex composition					
	All-Male (*n* = 150)		All-Female (*n* = 190)		Mixed-Sex (*n* = 272)	
	Wave 1	Wave 2	Wave 1	Wave 2	Wave 1	Wave 2
(a) Physical aggression						
*M* (*SD*)	1.60 (.91)	1.79 (.91)	1.54 (.88)	1.62 (.97)	1.78 (1.05)	1.68 (.98)
Missing	.3%	0%	3.2%	0%	4.2%	0%
*n* per category						
1	91	70	115	120	142	161
2	38	53	41	41	51	63
3	8	16	10	10	26	22
4	12	11	12	19	30	26
Actors change						
Decrease	19		30		49	
Increase	44		39		45	
Stable	86		109		155	
Missing	1		12		23	
(b) Prosociality						
*M* (*SD*)	1.44 (.73)	1.68 (.76)	1.78 (.64)	1.80 (.68)	1.87 (.79)	1.87 (.83)
Missing	.3%	0%	3.2%	0%	4.2%	0%
*n* per category						
1	99	69	55	61	84	98
2	39	66	112	111	125	127
3	6	9	6	13	28	32
4	5	6	5	5	12	15
Actors change						
Decrease	20		37		65	
Increase	54		41		58	
Stable	75		109		126	
Missing	1		12		23	
(c) Perceived Popularity						
*M* (*SD*)	3.48 (1.46)	3.61 (1.60)	3.28 (1.54)	3.51 (1.53)	3.61 (1.74)	3.59 (1.73)
Missing	.3%	0%	3.2%	0%	4.2%	0%
*n* per category						
1	15	20	28	26	37	55
2	16	24	24	25	39	25
3	59	19	56	31	52	25
4	19	39	34	69	29	93
5	21	28	12	12	40	20
6	19	20	24	27	52	54
Actors change						
Decrease	45		40		85	
Increase	56		75		95	
Stable	48		63		69	
Missing	1		12		23	

*n* per category refers to the number of participants within each category. Actors change refers to the number of participants who either decreased, increased or remained stable in physical aggression, prosociality, and perceived popularity between both time points

Furthermore, Table 1a, b shows the number of participants (actors) who increased, decreased or remained stable for physical aggression, prosociality, and perceived popularity. It appeared that participants were most likely to increase in aggression and prosocial behaviors in all-male classrooms. More than half of the participants across the three contexts displayed the same level of physical aggression and prosocial behavior over time. Popularity was more labile; across all three contexts only about a third of adolescents kept their popularity levels over time (see Table 1c).

The descriptive statistics for the friendship networks revealed that the density (i.e., the proportion of friendships relative to the potential maximum number of dyadic relations) in mixed-sex classrooms was lower compared with all-male and all-female classrooms (see Table 2). The Jaccard index showed that friendships were dynamic across all three contexts; about 20% of friendships remained stable, therefore allowing for a longitudinal social network analysis. No other large differences were found among the three contexts.

**Table 2:** Descriptive statistics for friendship networks

	Friendship networks					
	All-Male (*n* = 150)		All-Female (*n* = 190)		Mixed-Sex (*n* = 272)	
	Wave 1	Wave 2	Wave 1	Wave 2	Wave 1	Wave 2
Density^a^	.019	.023	.013	.014	.009	.011
Average degree^b^	2.84	3.44	2.46	2.61	2.55	2.96
Number of ties	421	513	451	472	680	784
Mutual ties	194	274	190	220	290	400
Asymmetric ties	426	464	448	440	626	616
Missing fraction	.013	.007	.035	.048	.018	.027
Tie changes						
Absence of tie (0 → 0)	21,236		32,729		69,793	
Creating tie (0 → 1)	335		310		467	
Resolving tie (1 → 0)	258		261		337	
Stable tie (1 → 1)	156		109		208	
Jaccard index^c^	.208		.160		.206	
Missing	2%		7%		4%	

^a^Density reflects the proportion of friendships relative to the total number of possible relations

^b^Average degree represents the average number of friendship nominations given

^c^Jaccard index indicates the proportion of stable relations from the total number of created, resolved, and stable relations

Bivariate correlations showed that prosociality was positively associated with perceived popularity in all three contexts: all-male (*r* = .38, *p* < .001), all-female (*r* = .33, *p* < .001), and mixed-sex (*r* = .36, *p* < .001). Physical aggression was associated with perceived popularity in all-female (*r* = .41, *p* < .001) and in mixed-sex (*r* = .24, *p* < .001) classrooms, but not in all-male (*r* = −.002, *p* = .98) classrooms. These results show that the function of physical aggression as a marker of perceived popularity differs across these contexts. Interestingly, physical aggression and prosociality were unrelated across all three contexts: all-male (*r* = −.11, *p* = .17), all-female (*r* = .07, *p* = .33), and mixed-sex (*r* = −.08, *p* = .21)..

### RSIENA Analyses

#### Structural Network Effects

Looking at the structural network effects among the three contexts revealed comparable findings. The negative density effect indicates that in all three contexts participants nominated less than half of their classmates as friends, reflecting the fact that participants could nominate a maximum of six peers as friends (see Table 3). Also, friendship nominations were reciprocal (positive reciprocity effect) and tended to be transitive, that is, friends of friends were likely to become friends too (transitive triplets effect).

**Table 3:** Results RSIENA analyses

	Friendship networks								
	All-Male (*n* = 150)			All-Female (*n* = 190)			Mixed-Sex (*n* = 272)^a^		
	*b*	*SE*	95% CI	*b*	*SE*	95% CI	*b*	*SE*	95% CI
(a) Network dynamics									
Structural network effects									
Density (outdegree)	−1.59*	.10	−1.79, −1.39	−2.38*	.20	−2.77, −1.99	−2.45*	.13	−2.70, −2.20
Reciprocity	1.47*	.16	1.16, 1.78	2.26*	.39	1.50, 3.02	1.60*	.26	1.09, 2.11
Transitive triplets	.29*	.06	.17, .41	.32*	.06	.20, .44	.21*	.04	.13, .29
Selection effects									
Physical aggression ego	.13	.12	−.11, .37	.11	.09	−.07, .29	−.07	.10	−.27, .13
Physical aggression alter	−.18	.10	−.38, .02	−.28*	.13	−.53, −.03	−.11	.09	−.29, .07
Physical aggression selection-similarity	.03	.11	−.19, .25	.09	.07	−.05, .23	.06	.06	−.06, .18
Prosocial ego	.04	.13	−.21, .29	−.27	.16	−.58, .04	−.11	.11	−.33, .11
Prosocial alter	.18	.10	−.02, .38	.32*	.13	.07, .57	.13	.11	−.09, .35
Prosocial selection-similarity	.15	.18	−.20, 050	−.14	.27	−.67, .39	.03	.10	−.17, .23
Perceived popularity ego	−.21*	.07	−.35, −.07	−.10	.07	−.24, .04	−.13*	.06	−.25, −.01
Perceived popularity alter	.11*	.06	−.01, .23	.16*	.07	.02, .30	.18*	.06	.06, .30
Perceived popularity selection-similarity	.06	.04	−.02, .14	.02	.03	−.04, .08	.08*	.03	.02, .14
(b) Behavior dynamics									
Behavioral tendencies									
Physical aggression linear	−.30	.20	−.69, .09	−1.17*	.20	−1.56, −.78	−1.03*	.14	−1.30, −.76
Prosociality linear	−.21	.23	−.66, .24	−.39	.36	−1.10, .32	−.34*	.12	−.58, −.10
Perceived popularity linear	−.03	.09	−.21, .15	−.02	.11	−.24, .20	−.03	.06	−.15, .09
Physical aggression quadratic	.07	.14	−.20, .34	.48*	.14	.21, .75	.34*	.10	.14, .54
Prosociality quadratic	−.15	.16	−.46, .16	−.76	.64	−2.01, .49	−.17	.13	−.42, .08
Perceived popularity quadratic	−.05	.05	−.15, .05	−.16	.09	−.34, .02	.03	.03	−.03, .09
Influence effects									
Physical aggression average alter	.19	.56	−.91, 1.29	.80	.54	−.26, 1.86	.35	.33	−.30, 1.00
Prosociality average alter	.20	.55	−.88, 1.28	1.70	1.85	−1.93, 5.33	.33	.43	−.51, 1.17
Perceived popularity average alter	.42*	.18	.07, .77	.61*	.30	.02, 1.20	.16*	.08	.00, .32

^a^Models for mixed-sex classrooms also included effects for sex alter, *b* = .02 (.13), *p* = .88; sex ego, *b* = .13(.15), *p* = .39; and same-sex, *b* = .78(.10), *p* < .001, in the network dynamics part of the model, revealing a strong tendency of participants to affiliate with same-sex peers

*p < .05

#### Selection Effects

Contrary to our expectations (Hypothesis 1a), the effect of physical aggression on nominating peers as friend over time (physical aggression ego effect) was not more pronounced in all-female classrooms than in all-male classrooms because it was nonsignificant across all contexts. With regard to the effect of physical aggression on being nominated as a friend (Hypothesis 1b), we found that aggressive adolescents were less likely to be nominated as friends one year later (negative physical aggression alter effect) only in all-female classrooms. Still, these effects did not structurally differ between contexts. This also holds for the selection-similarity effect for physical aggression (the likelihood of selecting as friends peers with similar aggression levels; Hypothesis 1c), which was nonsignificant in all three contexts. Together, these findings indicate that the role of physical aggression in shaping peer relations over time does not differ between all-male, all-female, and mixed-sex classrooms.

For prosociality, we found no strong evidence that prosocial behavior decreased the number of friendship nominations given over time (negative prosocial ego effect). This is contrary to our expectation that prosocial behavior would matter more for selection processes in male classrooms than in female classrooms (Hypothesis 2a). Furthermore, we found a trend that prosocial adolescents were more likely to receive friendship nominations (positive prosocial alter effect). Although this effect was only significant in all-female classrooms, it does not clearly differentiate between the three contexts as was expected (Hypothesis 2b). Similar to physical aggression, no selection-similarity was found for prosocial behavior in one of the three contexts (Hypothesis 2c). These findings suggest that the role of prosocial behavior in selection processes over time is almost similar across contexts.

Looking at the selection effects for perceived popularity, we found that in both all-male and mixed-sex settings, popular adolescents were more selective in nominating peers as friends (negative perceived popularity ego effect), whereas across the three classrooms contexts, more popular adolescents received more friendship nominations from their peers one year later (positive perceived popularity alter effect). Regarding selection-similarity, only in mixed-sex classrooms were friendships based on similar levels of perceived popularity levels (selection-similarity effect).

#### Behavioral Tendencies

The negative linear shape effect for physical aggression found in all-female and mixed-sex classrooms, and the negative prosocial linear shape effect in mixed-sex classrooms, showed that in these contexts the majority of students scored below the mean on these attributes. The positive quadratic shape effect for physical aggression observed in all-female and mixed-sex classrooms revealed a self-reinforcing effect, implying that adolescents who scored high in physical aggression became more aggressive over time, whereas adolescents at the lower extreme of the aggression scale became even less aggressive over time.

#### Influence Effects

Although we expected that influence processes for physical aggression (Hypothesis 1d) and prosociality (Hypothesis 2d) would differ between contexts, we found no influence effects for both behaviors in any of the contexts (physical aggression and prosocial average alter effect). The influence effect for perceived popularity was significant in all three contexts, indicating that perceived popularity of friends increased a person’s individual perceived popularity over time (perceived popularity average alter effect).

Comparing to what extent effects among the three contexts differed from each other statistically, we additionally calculated a *z*-difference score for all the separate estimates in our model comparing all-male with all-female and mixed-sex classrooms, as well as all-female classrooms with mixed-sex classrooms. These comparisons revealed that no significant difference emerged between the effects of interests of our study (see Online Supplementary material). That is, the strength of the alter-, ego-, selection-similarity, and influence effects for physical aggression, prosociality, and perceived popularity were similar across all three contexts.

## Discussion

In the present study we tested the roles of physical aggression and prosocial behavior in selection and influence processes across all-male, all-female, and mixed-sex classrooms. In doing so, we aimed to enhance our knowledge of how the development of behaviors and peer relations reflected in selection and influence processes were affected by gender-specific contexts. Building on earlier studies showing that physical aggression and prosociality were significant attributes in shaping adolescent’ friendships (Card et al. 2008; Crick and Grotpeter 1995; Martin and Halverson 1981; Rose and Rudolph 2006), we hypothesized that gender non-conforming behavior (physical aggression among female adolescents and prosocial behavior among male adolescents) would be particularly salient in sex-segregated contexts.

However, contrary to our hypothesis, we did not find that single-sex contexts made gender non-normative behavior more salient in friendship selection and influence processes. It appeared that the effects of physical aggression and prosociality were quite consistent across contexts. Regarding the selection dynamics, there was a general trend that physical aggression decreased and prosociality increased the likelihood of being nominated as a friend in all-male and particularly in all-female classrooms. Further, perceived popularity increased nominations received across all three contexts. In none of the contexts were children likely to select peers as friends who were similar in physical aggression or prosocialiy. Selection of friends who were similar in perceived popularity only played a role in mixed-sex settings.

Regarding behavioral dynamics, only perceived popularity was subject to peer influence processes across all three contexts, whereas physical aggression and prosocial behavior were not. Formal comparisons of estimates revealed that none of the effects of interests (ego, alter, selection-similarity, and influence effects for neither physical aggression and prosociality nor perceived popularity) reached significance. This clearly underlines that the effects of aggression and prosociality (as well as popularity) seem to play a similar role within different contexts.

Together these findings do not suggest systematic differences in the way physical aggression and prosociality, as well as perceived popularity, unfold in gender-specific contexts. Apparently, gender specificity in terms of behaviors with males being more aggressive and females being more prosocial (Rose and Rudolph 2006) do not translate differently in the developmental processes of selection and influence in all-female, all-male, and mixed-sex peer contexts. Moreover, the role of perceived popularity in friendship selection and influence processes seems to be fundamental across contexts, constituting a normative developmental process for males and females regardless of the peer group’s gender composition (see also Dijkstra et al. 2013; Marks et al. 2012).

These results suggest that gender differences in behaviors at the individual level might not be related in a straightforward manner to the gender specificity of certain contexts (Faris and Felmlee 2011). The social functions of aggression and prosocial behavior in shaping adolescents’ relationships appear to be remarkably similar. Thus, although there seems to be a gender-specific value or significance in these behaviors (Rose and Rudolph 2006), our findings reveal that it does not necessarily lead to differences in relational processes among young men and women.

One explanation might be that gender segregation remains within mixed-sex schools (Mehta and Strough 2009). This would imply that friendship formation and influence processes unfold within same-sex groups, as well as within mixed-sex groups. As a consequence, same-gender peers still function as the reference group, and behaviors are considered in the light of relations with same-gender peers, even in the presence of cross-gender peers (Mehta and Strough 2010).

It is worth noting the developmental phase that was assessed in our study. Physical aggression during adolescence might be widely disapproved by the peer culture because self-regulatory processes and the internalization of anti-aggressive social norms should have already been achieved. However, during earlier phases (i.e., late childhood), gender-specific behaviors might be fostered by peers without systematic rejection in the wider peer culture. Therefore, the present findings might not be replicable in younger populations.

From a different perspective, the present study constitutes an advance in the developmental and educational literature by applying social network analysis to gender-segregated educational contexts. The introduction of stochastic actor-based modeling as implemented in SIENA has yielded a rapidly growing number of studies on selection and influence processes among children and adolescents examining a wide range of behaviors and characteristics, such as smoking (Kiuru et al. 2010; Lakon et al. 2015), alcohol and drug use (DeLay et al. 2013; Mathys et al. 2013; Tucker et al. 2014), delinquency (Osgood et al. 2013), aggression (Sijtsema et al. 2010a, 2010b), bullying (Lodder et al. 2016), weapon carrying (Dijkstra et al. 2010), externalizing problems (Fortuin et al. 2015), prosocial behavior (Logis et al. 2013), and academic achievement (Gremmen et al. 2017). The methodological approach adopted in our study allowed describing longitudinal processes by assessing how peer relations interplay with individual physical aggression, prosocial behavior, and perceived popularity over time.

### Limitations and Future Research Directions

The present study has various limitations that should be acknowledged. First, in our study we only focused on physical aggression and prosociality. However, due to the traditionally assumed gender specificity of physical (male) and relational (female) aggression, both forms of aggression could be considered in further explorations of gender differences. Second, our networks were limited to the classroom, ignoring relations outside the classroom. Including these relations would explore whether specific behaviors are more or less prominent in peer relations between single-sex and mixed-sex groups within school. Future studies could overcome these limitations by including specific forms of aggression and other attributes within larger peer networks that might be relevant for adolescents’ friendships. Third, participants in our study were limited to nominate up to six classmates. Although unlimited number of nominations implies much more dispersion in the scores, we believe that we identified the most salient peers who display physical aggression and prosociality and who are considered popular. Fourth, we should be aware of the fact that in the absence of clear differences between contexts, this does not necessarily confirm that no difference exists (i.e., Type II error). Future research might further aim to unravel differences between contexts defined by sex. In line with this, our research was conducted in Chile rather than in the United States or Europe, in which peer relation research is predominantly conducted. To understand the universality of peer processes and put previous findings in cultural perspective, research might profit from including more understudied countries and cultures. Finally, our results should be taken with caution considering the size of our sample.

### Practice Implications

Our study contributes to practice professionals in educational contexts by highlighting the consistent relevance of physical aggression and prosociality as well as perceived popularity in gendered peer relations. Concretely, our findings show that adolescents’ friendships, and by implication peer relations in general, follow almost similar processes across gendered contexts. Interventions to promote prosocial behavior and to lessen aggressive behavior should be aware of the roles of these behaviors play in peer relations. In sum, the role and relevance of perceived popularity for adolescents’ relationships should not be underestimated.

### Conclusion

In the present study we tested the effects of physical aggression and prosocial behavior, while controlling for perceived popularity, focusing on potential differences caused by the gender-specific context in which they unfold. In doing so, we aimed to enhance our knowledge of how the development of behaviors and peer relations reflected in selection and influence processes are affected by the gendered context. Findings showed that friends’ selection and influence processes among adolescents are not specific to the gendered nature of the peer context, contributing to our understanding of the gender specificity and universality of these behaviors in peer relations across contexts.

## Electronic supplementary material

ESM 1 (DOCX 24 kb)
