# Supplementary material for: Friendship Selection and Influence Processes for Physical Aggression and Prosociality: Differences between Single-Sex and Mixed-Sex Contexts
Source: Sex Roles. 2017 Sep 13;78(9):625–36. doi: 10.1007/s11199-017-0818-z (PMC5897469; doi:10.1007/s11199-017-0818-z)
Supplement: Supplementary file 1 — (DOCX 24 kb) [file 11199_2017_818_MOESM1_ESM.docx]

Online supplement for Dijkstra, J. K., & Berger, C. (2017). Friendship selection and influence processes for physical aggression and prosociality: Differences between single-sex and mixed sex contexts. *Sex Roles*. Jan Kornelis Dijkstra, University of Groningen/ICS. Email: [jan.dijkstra@rug.nl](mailto:jan.dijkstra@rug.nl)

Table S1

*Results Comparison Estimates between All-Male and All-Female Contexts*

|  | Friendship networks | | | |  |
| --- | --- | --- | --- | --- | --- |
|  | All-Male (*n* = 150) | | All-Female (*n* = 190) | |  |
|  | *b* | *SE* | *b* | *SE* | Difference test |
|  | 1. **Network Dynamics** | | | |  |
| Structural network effects |  |  |  |  |  |
| Density (outdegree) | -1.59* | 0.10 | -2.38* | 0.20 | 5.64 |
| Reciprocity | 1.47* | 0.16 | 2.26* | 0.39 | -2.53 |
| Transitive triplets | 0.29* | 0.06 | 0.32* | 0.06 | -0.47 |
| Selection effects |  |  |  |  |  |
| Physical aggression ego | 0.13 | 0.12 | 0.11 | 0.09 | 0.16 |
| Physical aggression alter | -0.18 | 0.10 | -0.28* | 0.13 | 0.86 |
| Physical aggression selection-similarity | 0.03 | 0.11 | 0.09 | 0.07 | -0.52 |
| Prosocial ego | 0.04 | 0.13 | -0.27 | 0.16 | 1.99 |
| Prosocial alter | 0.18 | 0.10 | 0.32* | 0.13 | -1.20 |
| Prosocial selection-similarity | 0.15 | 0.18 | -0.14 | 0.27 | 1.15 |
| Perceived popularity ego | -0.21* | 0.07 | -0.10 | 0.07 | -1.47 |
| Perceived popularity alter | 0.11* | 0.06 | 0.16* | 0.07 | -0.77 |
| Perceived popularity selection-similarity | 0.06 | 0.04 | 0.02 | 0.03 | 0.98 |
|  | 1. **Behavior Dynamics** | | | |  |
| Behavioral tendencies |  |  |  |  |  |
| Physical aggression linear | -0.30 | 0.20 | -1.17* | 0.20 | 3.63 |
| Prosociality linear | -0.21 | 0.23 | -0.39 | 0.36 | 0.50 |
| Perceived popularity linear | -0.03 | 0.09 | -0.02 | 0.11 | -0.10 |
| Physical aggression quadratic | 0.07 | 0.14 | 0.48* | 0.14 | -2.57 |
| Prosociality quadratic | -0.15 | 0.16 | -0.76 | 0.64 | 1.07 |
| Perceived popularity quadratic | -0.05 | 0.05 | -0.16 | 0.09 | 1.89 |
| Influence effects |  |  |  |  |  |
| Physical aggression average alter | 0.19 | 0.56 | 0.80 | 0.54 | -0.72 |
| Prosociality average alter | 0.20 | 0.55 | 1.70 | 1.85 | -0.38 |
| Perceived popularity average alter | 0.42* | 0.18 | 0.61* | 0.30 | -0.70 |

Table S2

*Results Comparison Estimates between All-Male and Mixed-Sex Contexts*

|  | Friendship networks | | | |  |
| --- | --- | --- | --- | --- | --- |
|  | All-Male (*n* = 150) | | Mixed-Sex (*n* = 272) | |  |
|  | *b* | *SE* | *b* | *SE* | Difference test |
|  | 1. **Network Dynamics** | | | |  |
| Structural network effects |  |  |  |  |  |
| Density (outdegree) | -1.59* | 0.10 | -2.45* | 0.13 | 7.36 |
| Reciprocity | 1.47* | 0.16 | 1.60* | 0.26 | -0.57 |
| Transitive triplets | 0.29* | 0.06 | 0.21* | 0.04 | 1.30 |
| Selection effects |  |  |  |  |  |
| Physical aggression ego | 0.13 | 0.12 | -0.07 | 0.10 | 1.54 |
| Physical aggression alter | -0.18 | 0.10 | -0.11 | 0.09 | -0.65 |
| Physical aggression selection-similarity | 0.03 | 0.11 | 0.06 | 0.06 | -0.26 |
| Prosocial ego | 0.04 | 0.13 | -0.11 | 0.11 | 1.06 |
| Prosocial alter | 0.18 | 0.10 | 0.13 | 0.11 | 0.45 |
| Prosocial selection-similarity | 0.15 | 0.18 | 0.03 | 0.10 | 0.63 |
| Perceived popularity ego | -0.21* | 0.07 | -0.13* | 0.06 | -1.09 |
| Perceived popularity alter | 0.11* | 0.06 | 0.18* | 0.06 | -1.10 |
| Perceived popularity selection-similarity | 0.06 | 0.04 | 0.08* | 0.03 | -0.49 |
|  | 1. **Behavior Dynamics** | | | |  |
| Behavioral tendencies |  |  |  |  |  |
| Physical aggression linear | -0.30 | 0.20 | -1.03* | 0.14 | 3.32 |
| Prosociality linear | -0.21 | 0.23 | -0.34* | 0.12 | 0.53 |
| Perceived popularity linear | -0.03 | 0.09 | -0.03 | 0.06 | 0.00 |
| Physical aggression quadratic | 0.07 | 0.14 | 0.34* | 0.10 | -1.80 |
| Prosociality quadratic | -0.15 | 0.16 | -0.17 | 0.13 | 0.11 |
| Perceived popularity quadratic | -0.05 | 0.05 | 0.03 | 0.03 | -1.57 |
| Influence effects |  |  |  |  |  |
| Physical aggression average alter | 0.19 | 0.56 | 0.35 | 0.33 | -0.24 |
| Prosociality average alter | 0.20 | 0.55 | 0.33 | 0.43 | -0.18 |
| Perceived popularity average alter | 0.42* | 0.18 | 0.16* | 0.08 | 1.39 |

Table S3

*Results Comparison Estimates between All-Female and Mixed-Sex Contexts*

|  | Friendship networks | | | |  |
| --- | --- | --- | --- | --- | --- |
|  | All-Female (*n* = 190) | | Mixed-Sex (*n* = 272) | |  |
|  | *b* | *SE* | *b* | *SE* | Difference test |
|  | 1. **Network Dynamics** | | | |  |
| Structural network effects |  |  |  |  |  |
| Density (outdegree) | -2.38* | 0.20 | -2.45* | 0.13 | 0.32 |
| Reciprocity | 2.26* | 0.39 | 1.60* | 0.26 | 1.44 |
| Transitive triplets | 0.32* | 0.06 | 0.21* | 0.04 | 1.79 |
| Selection effects |  |  |  |  |  |
| Physical aggression ego | 0.11 | 0.09 | -0.07 | 0.10 | 1.80 |
| Physical aggression alter | -0.28* | 0.13 | -0.11 | 0.09 | -1.23 |
| Physical aggression selection-similarity | 0.09 | 0.07 | 0.06 | 0.06 | 0.41 |
| Prosocial ego | -0.27 | 0.16 | -0.11 | 0.11 | -0.93 |
| Prosocial alter | 0.32* | 0.13 | 0.13 | 0.11 | 1.34 |
| Prosocial selection-similarity | -0.14 | 0.27 | 0.03 | 0.10 | -0.61 |
| Perceived popularity ego | -0.10 | 0.07 | -0.13* | 0.06 | 0.41 |
| Perceived popularity alter | 0.16* | 0.07 | 0.18* | 0.06 | -0.27 |
| Perceived popularity selection-similarity | 0.02 | 0.03 | 0.08* | 0.03 | -1.94 |
|  | 1. **Behavior Dynamics** | | | |  |
| Behavioral tendencies |  |  |  |  |  |
| Physical aggression linear | -1.17* | 0.20 | -1.03* | 0.14 | -0.64 |
| Prosociality linear | -0.39 | 0.36 | -0.34* | 0.12 | -0.13 |
| Perceived popularity linear | -0.02 | 0.11 | -0.03 | 0.06 | 0.09 |
| Physical aggression quadratic | 0.48* | 0.14 | 0.34* | 0.10 | 0.93 |
| Prosociality quadratic | -0.76 | 0.64 | -0.17 | 0.13 | -0.90 |
| Perceived popularity quadratic | -0.16 | 0.09 | 0.03 | 0.03 | -2.09 |
| Influence effects |  |  |  |  |  |
| Physical aggression average alter | 0.80 | 0.54 | 0.35 | 0.33 | 0.69 |
| Prosociality average alter | 1.70 | 1.85 | 0.33 | 0.43 | 0.67 |
| Perceived popularity average alter | 0.61* | 0.30 | 0.16* | 0.08 | 1.47 |
